# The La antigen is over-expressed in lung cancer and is a selective dead cancer cell target for radioimmunotherapy using the La-specific antibody APOMAB®

**DOI:** 10.1186/2191-219X-4-2

**Published:** 2014-01-04

**Authors:** Alexander H Staudacher, Fares Al-Ejeh, Cara K Fraser, Jocelyn M Darby, David M Roder, Andrew Ruszkiewicz, Jim Manavis, Michael P Brown

**Affiliations:** 1 Translational Oncology Laboratory, Centre for Cancer Biology, SA Pathology, Adelaide, Australia; 2 School of Pharmacy and Medical Science, University of South Australia, Adelaide, Australia; 3 Signal Transduction Laboratory, Queensland Institute of Medical Research, Brisbane, Australia; 4 Cancer Epidemiology and Population Health, School of Population Health, University of South Australia, Adelaide, Australia; 5 Gastroenterology Research Laboratory, Centre for Cancer Biology, SA Pathology, Adelaide, Australia; 6 Centre for Neurological Diseases, SA Pathology, Adelaide, Australia; 7 Cancer Clinical Trials Unit, Royal Adelaide Hospital, Adelaide, Australia; 8 School of Medicine, University of Adelaide, Adelaide, Australia; 9 Current affiliation: Preclinical Imaging and Research Laboratories, South Australian Health and Medical Research Institute, Adelaide, Australia; 10 Experimental Therapeutics Laboratory, Level 4 Hanson Institute Building North, Royal Adelaide Hospital, North Terrace, Adelaide, SA, 5000, Australia

**Keywords:** APOMAB®, La/SSB antigen, Lewis lung carcinoma, PARP inhibitor, Lutetium-177, Bystander killing

## Abstract

**Background:**

The lupus-associated (La)-specific murine monoclonal antibody DAB4 (APOMAB®) specifically binds dead cancer cells. Using DAB4, we examined La expression in human lung cancer samples to assess its suitability as a cancer-selective therapeutic target. We evaluated the safety and effectiveness of radioimmunotherapy (RIT) using DAB4 radiolabeled with Lutetium-177 (^177^Lu) in the murine Lewis Lung (LL2) carcinoma model, and determined whether combining RIT with DNA-damaging cisplatin-based chemotherapy, a PARP inhibitor (PARPi), or both alters treatment responses.

**Methods:**

The expression of La mRNA in human lung cancer samples was analysed using the online database Oncomine, and the protein expression of La was examined using a TissueFocus Cancer Survey Tissue Microarray. The binding of DAB4 to cisplatin-treated LL2 cells was assessed *in vitro*. LL2 tumour-bearing mice were administered escalating doses of ^177^Lu-DAB4 alone or in combination with chemotherapy, and tumour growth and survival measured. Biodistribution analysis was used to determine tissue uptake of ^177^Lu-DAB4 or its isotype control (^177^Lu-Sal5), when delivered alone or after chemotherapy. PARPi (rucaparib; AG-014699) was combined with chemotherapy and the effects of combined treatment on tumour growth, tumour cell DNA damage and death, and intratumoural DAB4 binding were also analysed. The effect of the triple combination of PARPi, chemotherapy and ^177^Lu-DAB4 on tumour growth and survival of LL2 tumour-bearing mice was tested.

**Results:**

La was over-expressed at both mRNA and protein levels in surgical specimens of human lung cancer and the over-expression of La mRNA conferred a poorer prognosis. DAB4 bound specifically to cisplatin-induced dead LL2 cells *in vitro*. An anti-tumour dose response was observed when escalating doses of ^177^Lu-DAB4 were delivered *in vivo*, with supra-additive responses observed when chemotherapy was combined with ^177^Lu-DAB4. Combining PARPi with chemotherapy was more effective than chemotherapy alone with increased tumour cell DNA damage and death, and intratumoural DAB4 binding. The combination of PARPi, chemotherapy and ^177^Lu-DAB4 was well-tolerated and maximised tumour growth delay.

**Conclusions:**

The La antigen represents a dead cancer cell-specific target in lung cancer, and DAB4 specifically targeted tumour tissue *in vivo*, particularly after chemotherapy. Tumour uptake of DAB4 increased further after the combination of PARPi and chemotherapy, which generated new dead tumour cell-binding targets. Consequently, combining ^177^Lu-DAB4 with PARPi and chemotherapy produced the greatest anti-tumour response. Therefore, the triple combination of PARPi, chemotherapy and RIT may have broad clinical utility.

## Background

Lung cancer, particularly the common non-small cell lung cancer (NSCLC) variant, is one of the major contributors to cancer death worldwide [1]. Moreover, the global incidence of NSCLC continues to rise, particularly among females in developed economies, and generally among persons in emerging economies. Metastatic NSCLC is typically incurable, with standard first-line treatment for good-performance status patients being platinum-based doublet chemotherapy which is believed to have reached an efficacy plateau. More recently, the identification of molecular lesions in NSCLC, such as activating mutations of the epidermal growth factor receptor (*EGFR*) gene and fusions of the anaplastic lymphoma kinase (*ALK*) gene, has yielded targets for small-molecule kinase inhibitors, which often produce regressions and durable tumour control [2]. Nevertheless, the need for innovative improvements to platinum-based chemotherapy remains because kinase inhibitors do not cure, and other clinically actionable molecular lesions are found in approximately half of NSCLC cases [3]. Furthermore, post-operative cisplatin-based chemotherapy is the standard treatment for good-performance status patients with completely resected early-stage NSCLC because chemotherapy confers a 5-year absolute survival benefit of 5.4% [4].

Targeting tumours with therapeutic monoclonal antibodies (mAbs) has proven to be an effective approach to cancer therapy although its clinical effectiveness has been more limited in NSCLC with the vascular endothelial growth factor-neutralising mAb, bevacizumab, the only approved therapy. Hence, lung cancer is relatively bereft of cell-surface targets for antibody therapies. The anti-tumour potency of mAbs can be improved by arming mAbs with radionuclides and cytotoxic drugs, creating the new therapeutic modalities of radioimmunotherapy (RIT) and antibody drug conjugates, respectively. Although armed mAbs usually target tumour cell-surface antigens, they can also elicit bystander killing of surrounding, antigen-negative tumour cells [5,6]. Successful β-emitting RIT has led to the approval of two agents for treating CD20-positive B-cell lymphomas, although RIT for non-haematological malignancies has been limited by heterogeneous antigen expression and radio-resistance among other factors reviewed by [7].

The lupus-associated (La) antigen, also known by the HUGO Gene Nomenclature Committee approved name of Sjögren Syndrome B (SSB), is an abundant, essential, and ubiquitously expressed ribonucleoprotein that is revealed preferentially in dead tumour cells because La is over-expressed in malignancy [8-10] and dead tumour cells are inefficiently cleared *in vivo* unlike dead normal cells [10,11]. The La-specific mAb, DAB4 (APOMAB®), targets dead tumour cells *in vivo*[12], particularly after DNA-damaging anti-cancer treatment [10,11]. Using DAB4 labeled with the β-emitter Yttrium-90 (^90^Y), we have tested the concept of targeting dead tumour cells as a way to deliver RIT to surrounding viable tumour cells in murine syngraft and human xenograft models. Compared to DAB4-directed RIT alone, prior treatment with DNA-damaging cytotoxic chemotherapy resulted in supra-additive anti-tumour responses, with the quality of the responses ranging from significant tumour growth delay in carcinoma models [11] to sustained complete regression in a lymphoma model [13].

The safety and efficacy of RIT in carcinoma models may be improved by altering the radionuclide used for RIT and by combining RIT with radio-sensitising drugs or small molecule enzyme inhibitors [13]. Since carcinomas often evolve because of defective DNA damage responses [14], DNA repair inhibitors represent an attractive target for sensitising resistant cancer cells to treatment [15]. One such target is the family of Poly ADP ribose polymerases (PARP), three of which (PARP-1, PARP-2, PARP-3) are involved in the DNA damage response to DNA single strand breaks (SSB) [16]. Unrepaired SSB can result in collapsed replication forks and formation of double-strand breaks (DSB) which are repaired by homologous recombination (HR). Some tumour types have defective HR repair and rely solely on SSB repair through PARP signalling, and targeting PARP itself is sufficient to cause synthetic lethality reviewed by [17]. PARPi are also chemo- and radio-sensitising agents which reduce DNA repair capacity, allowing accumulation of DNA damage which can lead to cell death [18]. Additionally, some PARP inhibitors have vasodilative properties [19], which contribute to increased tumour sensitisation to radiation [18,20] and some [21,22] but not [23] all chemotherapeutics.

In this study, we analysed expression of La mRNA in normal and malignant lung tissues and the expression of La protein in surgical specimens from lung cancer patients. We used ^177^Lu-DAB4 with or without radio-sensitising chemotherapy for treating mice harbouring the well-characterised chemo- and radio-resistant Lewis Lung (LL2) tumour. We hypothesised that adding a PARPi to the treatment regimen would sensitise tumour cells to chemotherapy and generate more intratumoural dead cell targets for DAB4 to bind. The increased tumour accumulation of ^177^Lu-DAB4 together with continued PARPi treatment would further sensitise tumour cells to ^177^Lu-derived β-emissions, resulting in greater anti-tumour activity.

## Methods

### Data-mining of La mRNA expression in lung cancer

The online cancer database Oncomine version 4.4.3 (Compendia Bioscience Inc., Ann Arbor, MI, USA) was used to examine the mRNA expression of La in normal versus cancerous lung tissue, with a *p* value significance threshold set at <10^-4^.

### Cell culture and antibody production

LL2 cells were cultured in RPMI-1640 (Sigma-Aldrich, St. Louis, MO, USA) with 5% FCS (Bovogen Biologicals, Keilor East, Victoria, Australia). DAB4 is a subclone of the anti-La 3B9 hybridoma originated by Dr Michael Bachmann [24] which was selected on the basis of higher binding to a defined epitope of the La antigen. 3B9 was the gift of Prof. Tom Gordon, SA Pathology, Flinders Medical Centre, Adelaide, Australia. DAB4 and the isotype-specific control mAb (Sal5) were produced and purified as previously described [10,25,26].

### Detection of La protein in human tumour samples

A TissueFocus Cancer Survey Tissue Microarray (Origene, Rockville, MD, USA) was dewaxed and endogenous peroxidase activity was blocked with 0.5% hydrogen peroxide in methanol. After washing in PBS, heat-induced antigen retrieval using citrate buffer (pH 6) was performed. The slide was allowed to cool and washed twice with PBS followed by blocking with 3% normal human serum. The slide was incubated with 0.6 μg/mL DAB4 overnight at room temperature, followed by biotinylated horse anti-mouse IgG (Vector Laboratories, Burlingame, CA, USA) and strepavidin conjugated horseradish peroxidase (Thermo-Fisher, Waltham, MA, USA). To visualise antibody binding, the microarray was incubated with 3′-diaminobenzide and counterstained with Mayer’s Haematoxylin.

### Antibody binding to LL2 cells

LL2 cells (10^5^) were treated with varying concentrations of cisplatin for 48 h, collected, washed with FACS buffer (0.5% BSA, 0.04% sodium azide, PBS) and incubated with 10 μg/mL DAB4 or Sal5 for 15 min. Cells were washed and incubated with 2 μg/mL goat anti-mouse Alexa-488 (Life Technologies, Carlsbad, CA, USA) for 15 min, washed further, incubated with 1 μg/mL DAPI (Sigma-Aldrich) and analysed by flow cytometry (FACScanto flow cytometer, BD Biosciences, Franklin Lakes, NJ, USA).

### LL2 tumour model

All animal experiments were approved by the SA Pathology Animal Ethics Committee, Adelaide, and conducted following institutional ethical guidelines. Six- to 8-week-old female C57Bl/6 mice were injected subcutaneously in the right flank with 10^6^ LL2 cells. Tumour size was measured using electronic calipers and tumour volume determined using the calculation: tumour volume *=* (*a*^*2*^ *× b*)*/*2, where *a* is the shortest diameter and *b* is the longest diameter of the tumour. Treatment commenced when tumours reached 30 to 50 mm^3^. Mice were monitored daily using a clinical record sheet with points allocated for physical observations such as visible tumour, ruffled coat, hunched posture, reluctance to move, diarrhoea, squealing when handled and weight loss. Body weight and tumour volume were measured every 2 days in the first week of treatment and daily thereafter. Mice were humanely euthanized when a clinical score of 5 was reached, weight loss was >15% (*cf.* day 1), or tumour volume was >600 mm^3^.

### Treatment of tumour-bearing mice

Tumour-bearing mice were treated intravenously with 50 mg/kg gemcitabine (Hospira, Melbourne, VIC, Australia) on days 1 and 2 and 2.5 mg/kg cisplatin (Hospira) on day 1. DAB4 and Sal5 were conjugated to the bi-functional chelator DOTA-NHS (Macrocyclics, Dallas, TX, USA) as previously described [11] and radiolabeled with ^177^Lu (PerkinElmer, Waltham, MA, USA). Radioimmunoconjugates were administered intravenously on day 3. The specific activity of radioimmunoconjugates ranged from 95 to 130 MBq/mg with >97% incorporation of ^177^Lu as determined by instant thin layer chromatography. The PARPi inhibitor Rucaparib (AG-014699; Selleck Chemicals, Houston, TX, USA) was diluted in 5% D-glucose in PBS for intraperitoneal injection at 1 or 2 mg/kg and administered daily on days 1 to 5, 30 min before chemotherapy or RIT. For *in vivo* antibody binding analysis, DAB4 was biotinylated with EZ-Link NHS-Biotin (Thermo Fisher) following manufacturer’s instructions. One hundred micrograms of biotin-DAB4 was administered 24 h after chemotherapy.

### Tissue biodistribution studies

Mice were euthanized 24 h after RIT administration, tissues were collected and weighed and radioactivity was measured using a Wallac 2470 wizard^2^ gamma counter (PerkinElmer) with peak detection set at 208 keV. Radioactivity in the organs was normalized to the weight of the organ and the accumulation was calculated as the percentage of radioactivity per gram over the radioactivity of the injected dose of ^177^Lu-DOTA immunoconjugates (%ID/g). High-resolution digital autoradiography was performed on 4-μm tumour sections using a MicroImager (Biospace Lab, Paris, France).

### Ex vivo analysis of LL2 tumours

Mice were euthanized 24 h after administration of biotin-DAB4, tumours were removed and snap frozen in OCT cryoprotectant (Sakura Finetek, Torrance, CA, USA). Sections were fixed with 10% neutral-buffered formalin, washed with PBS, blocked with 5% BSA with 0.3% Triton-X 100 (Sigma-Aldrich), washed and incubated overnight with rabbit anti-phospho-histone H2AX (Ser139) antibody (1:300 dilution; Cell Signaling Techology, Danvers, MA, USA). Sections were washed and incubated with donkey anti-rabbit IgG NL557 (1:200 dilution; R and D systems, Minneapolis, MN, USA). To detect biotin-DAB4, sections were concurrently incubated with strepavidin-Alexa Fluor-488 (1:400; Life Technologies). Cell death was detected using the ApopTag cell death detection kit (Millipore, Billerica, MA, USA) following manufacturer’s instructions. Sections were stained with 1 μg/mL DAPI, and examined using an Olympus IX71 microscope with CellSens Standard (v1.6) software and analysed using ImageJ (v1.45) software (National Institute of Health, Bethesda, MD, USA).

### Statistical analysis

Measurements of association between La mRNA expression and lung cancer outcomes [27] were performed using WINPEPI software [28]. Tumour doubling times were calculated as previously described [11]. Statistical analyses were performed using GraphPad Prism (v5.0) software, with data shown as mean ± SEM. Unless otherwise stated, intergroup comparisons were made by analysis of variance (ANOVA, Bonferroni post-hoc test). Kaplan-Meier median survival curves were compared using log-rank (Mantel-Cox) test.

## Results

### La is over-expressed at the mRNA level in lung cancer and exhibits strong immunostaining patterns in lung cancer tissue

**Figure 1:**
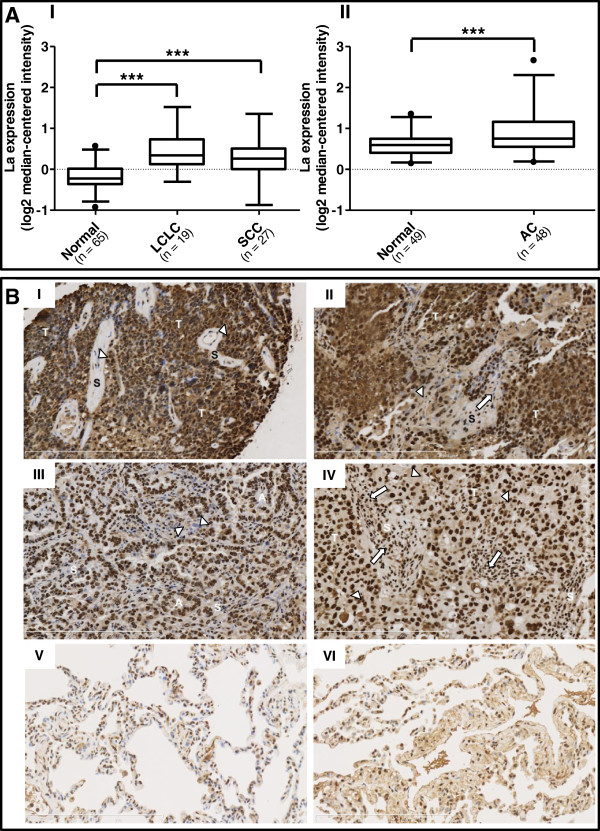
**Expression of La mRNA and protein in lung cancer. (A)** The expression of La mRNA in normal compared to lung cancer samples was examined using the online database Oncomine. Two studies showed increased La mRNA expression in lung cancer samples compared to normal lung tissue. Data was taken from (I) Hou et al.’s study [30] and (II) Landi et al*.*’s study [29]. Data are shown with 95% confidence intervals and circles representing outliers. LCLC, large cell lung cancer; SCC, squamous cell lung cancer; AC, adenocarcinoma. *** *p* < 0.001. **(B)** Photomicrographs of DAB4 staining pattern in sections of resected human lung tumours. TissueFocus Cancer Survery Tissue Microarray (Origene) was examined via immunohistochemistry for DAB4 expression. (I) Poorly differentiated squamous cell carcinoma of bronchus (case No. 0000019032): tumour cells (T); hypocellular stroma (S) with variable DAB4 expression (evident as brown staining) of normal cells including endothelial cells and other stromal cells (arrowheads). (II) Adenosquamous carcinoma (case No. 0000016518): predominantly basaloid squamous cell carcinoma (T) with prominent DAB4-stained nucleoli (arrowheads); desmoplastic stromal reaction (S) with both DAB4-stained and unstained nuclei of stromal cells (arrow). (III) Poorly differentiated adenocarcinoma (case No. 0000008378): acinar structures (A) are evidently interweaved with a hypercellular stroma (S). Some malignant cells were not stained with DAB4 (arrowheads). (IV) Poorly differentiated lung adenocarcinoma (case No. 0000016267): the high-grade malignant cells (T) are vacuolated and have weak non-specific cytoplasmic staining with DAB4 (arrowheads); a desmoplastic stromal reaction (S) with DAB4-stained lymphoplasmacytic infiltrates (arrows). (V and VI) Normal lung tissue (case No. 0000000144 and 0000006853) showed reduced, predominantly nuclear DAB4 staining. All sections are shown at × 20 magnification, and scale bars are indicated.

We used the Oncomine online gene expression database to examine the expression of La mRNA in human lung cancer samples compared to normal lung tissue. Two studies [29,30] demonstrated a significant 1.5-fold and 1.3-fold increase in La mRNA expression in large cell lung carcinoma (LCLC) and squamous cell lung carcinoma (SCC), respectively (Figure 1A (part I)) and 1.2-fold increase in lung adenocarcinoma (AC) compared to normal lung samples (Figure 1A (part II)). In a further analysis, there was a higher death rate in patients in whom surgical specimens of NSCLC had ≥2-fold increased expression of La mRNA compared to those whose tumours had <2-fold increased expression of La mRNA (Table 1), with a rate ratio (95% CI) of 1.27 (1.07, 1.50; *p* = 0.008). This ratio was very similar when adjusting for nodal status in a Mantel-Haenszel analysis at 1.27 (1.08, 1.49), and heterogeneity testing showed similarly elevated rate ratios for both the node-positive and node-negative cases (*p* = 0.806).

**Table 1:** Survival status of resected NSCLC patients according to La mRNA expression levels

**La expression (compared to healthy tissue)**	**Survival status**		**Number per group**
	**Dead**	**Alive**	
>2-fold increase	103 (61%)	65 (39%)	168
<2-fold increase	133 (48%)	142 (52%)	275

The mRNA expression of La and survival status of patients with resected NSCLC was examined using Oncomine, with data originally derived from [27].

The expression of La protein in lung cancer samples was also investigated by immunohistochemistry using a cancer tissue microarray stained with the La-specific mouse mAb, DAB4 and scored by a pathologist. Only nuclear staining with DAB4 was considered positive and scoring was based on staining intensity (weak, moderate or strong) and percentage of positively stained cells. In the 10 lung cancer sections, all demonstrated strong staining; in two sections, >95% tumour cells were positive, and in the remaining eight sections, >99% tumour cells were positive. In the 5 normal lung tissue sections, approximately 50% cell nuclei had weak to moderate DAB4 staining, mainly belonging to pneumocytes and endothelial, inflammatory and stromal cells. As illustrated in Figure 1B (parts I to IV), DAB4 stained nuclei of tumour cells more intensely than the nuclei of some tumour stromal cells. The most intense intranuclear staining of DAB4 was found within the nucleoli, which featured prominently in many malignant cells. In comparison, only moderate nuclear staining of DAB4 was observed among the epithelial and stromal cells of normal lung tissues (Figure 1B (parts V and VI)).

### DAB4 binding to dead LL2 cells in vitro

**Figure 2:**
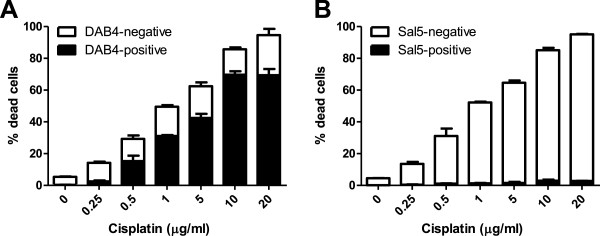
**DAB4 binding to dead LL2 cells *in vitro*.** LL2 cells were treated with cisplatin for 48 h and **(A)** DAB4 binding or **(B)** Sal5 binding to dead cells was examined by flow cytometry. *n* = 3.

The murine lung cancer LL2 cell line was treated *in vitro* with escalating doses of cisplatin for 48 h and binding of DAB4 or its isotype control mAb (Sal5) to dead (DAPI^+^) cells was examined. Cisplatin treatment resulted in a dose-dependent increase in cell death (Figure 2). As the percentage of dead cells increased, so did the proportion of dead, DAB4-bound cells (Figure 2A), with >80% of the dead cell population being bound by DAB4 after treatment with 10 μg/mL cisplatin. Conversely, only minimal binding of Sal5 (<3%) to dead cisplatin-treated cells was observed (Figure 2B).

### Treatment with ^177^Lu-DAB4 alone and in combination with chemotherapy

**Figure 3:**
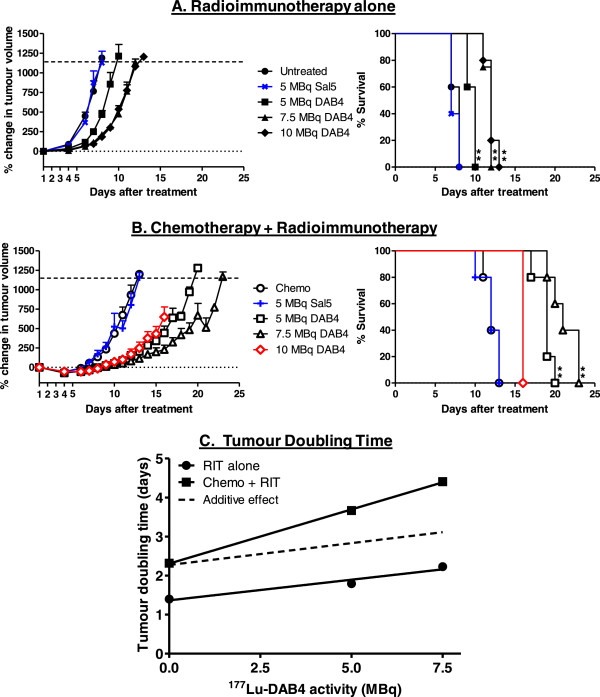
**Anti-tumour response of RIT alone or in combination with chemotherapy.** LL2 tumour bearing mice were left untreated, or treated with chemotherapy (chemo) and/or RIT (^177^Lu-DAB4 or ^177^Lu-Sal5). **(A)** Left panel, percentage change in tumour volume after treatment. Right panel, Kaplan-Meier survival analysis, ** *p* < 0.01 compared to untreated mice, *n* = 5. **(B)** Comparison of the anti-tumour effect of escalating doses of RIT when delivered after chemotherapy. Left panel, percentage change in tumour volume after treatment. Red symbols = toxic dose. Right panel, Kaplan-Meier survival analysis, ** *p* < 0.01 compared to mice given chemotherapy alone, *n* = 5. **(C)** Mean tumour doubling times derived from tumour growth curves are displayed as a function of RIT dose. Standard errors for data points range from 0.03 to 0.15, and are too small to be evident as error bars. Data shows that the combination of chemotherapy and RIT is supra-additive because the slopes of the two lines are significantly different (*p* = 0.02; analysis of covariance).

Next, we investigated if ^177^Lu-DAB4 exhibited anti-tumour activity that was similar to what we had previously shown with ^90^Y-DAB4 [11] in the LL2 syngraft model. As a monotherapy, ^177^Lu-DAB4 reduced tumour growth and significantly increased median survival time (MST) from 8 days for untreated mice to 10, 12 and 12 days after 5, 7.5 and 10 MBq ^177^Lu-DAB4, respectively (Figure 3A). As administering radiolabeled DAB4 after chemotherapy promotes tumour accumulation compared to simultaneous administration [12], ^177^Lu-DAB4 was given to LL2-tumour bearing mice 24 h after completion of chemotherapy (consisting of 50 mg/kg gemcitabine on days 1 and 2 and 2.5 mg/kg cisplatin on day 1; Figure 3B), which is the peak time for chemotherapy-induced tumour cell death [11]. The combination of chemotherapy and 5 or 7.5 MBq ^177^Lu-DAB4 further delayed tumour growth and significantly increased MST to 19 and 21 days, respectively, compared to chemotherapy alone (MST 12 days). The combination of chemotherapy and 10 MBq ^177^Lu-DAB4 was not tolerated, and the entire cohort was euthanized 16 days after treatment because 60% of the treatment group had a clinical score of 5. None of mice in the remaining treatment groups had evident toxicity, with only minimal and transient body weight loss observed (Additional file 1: Figure S1) and a clinical score of no more than 2. ^177^Lu-Sal5 alone or with chemotherapy was not effective (Figure 3A,B). Tumour doubling times (TDT) for treatment groups were determined and showed that while ^177^Lu-DAB4 alone increased TDT linearly with dose, the combination of chemotherapy and ^177^Lu-DAB4 resulted in a supra-additive response (Figure 3C). A similar supra-additive response was also observed when chemotherapy was combined with lower activities of ^177^Lu-DAB4, a result which mirrored our previous findings with ^90^Y-DAB4 (Additional file 2: Figure S2).

### Biodistribution of DAB4 in LL2 tumours

**Figure 4:**
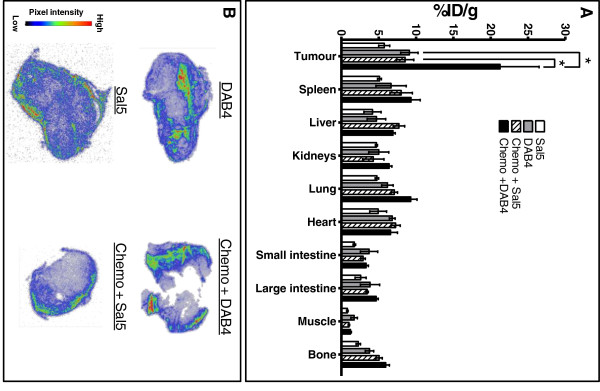
**Biodistribution of **^**177**^**Lu-DAB4 in LL2 tumour-bearing mice.** LL2 tumour-bearing mice were given RIT (^177^Lu-DAB4 or ^177^Lu-Sal5) alone or after chemotherapy (chemo), and **(A)** Tissue accumulation of radioactivity was assayed. * *p* < 0.05 (two-way ANOVA), *n* = 4. **(B)** The same tumours were sectioned, and imaged using a MicroImager.

The tissue distribution of ^177^Lu-DAB4 and ^177^Lu-Sal5 in LL2 tumour-bearing mice was examined to confirm whether the therapeutic response of ^177^Lu-DAB4 was associated with tumour-selective uptake of the radioimmunoconjugate. When delivered alone, ^177^Lu-DAB4 tumour accumulation was 1.6-fold higher than ^177^Lu-Sal5, with chemotherapy further increasing ^177^Lu-DAB4 tumour accumulation 2.5-fold compared to mice treated with chemotherapy and ^177^Lu-Sal5 (Figure 4A). Chemotherapy did not significantly affect the biodistribution of ^177^Lu-DAB4 in normal tissues, indicating that ^177^Lu-DAB4 specifically targeted tumour tissue after chemotherapy. β-microimaging of tumour sections showed heterogeneous uptake of ^177^Lu-DAB4 which was increased after chemotherapy (Figure 4B).

### PARPi increases the anti-tumour activity of chemotherapy in vivo

**Figure 5:**
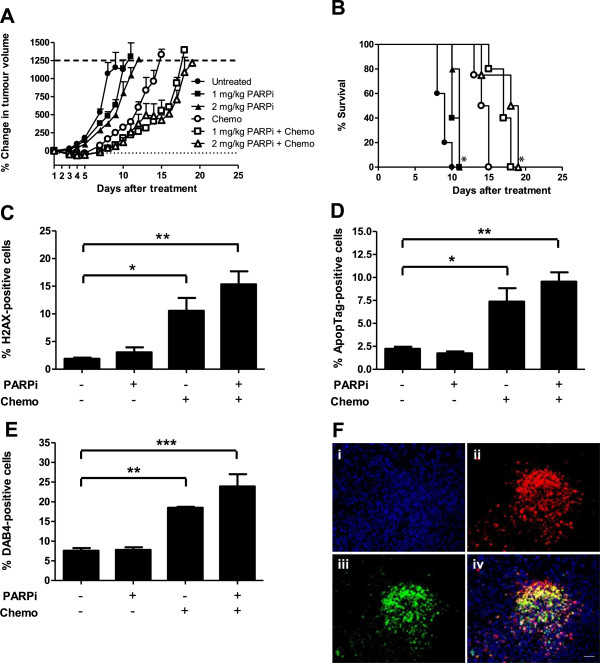
**Combination of PARPi with chemotherapy increases tumour growth delay, DNA damage, tumour cell death, and tumour uptake of DAB4 *in vivo*.** LL2 tumour-bearing mice were administered PARPi alone or with chemotherapy (chemo). **(A)** Percentage change in tumour volume after treatment **(B)** Kaplan-Meier survival analysis. * *p* < 0.05 compared to untreated or chemotherapy alone, *n* = 4 to 5. Tumour sections were examined for **(C)** γ-H2AX, **(D)** cell death (ApopTag+) and **(E)** DAB4 binding. * *p* < 0.05, ** *p* < 0.01, *** *p* <0.001, *n* = 3. **(F)** Shows (i) DAPI-counterstained tumour section with detection of (ii) cell death (ApopTag+), and (iii) biotinylated DAB4 with merged images shown in (iv; yellow = co-localisation of DAB4 with dead cells). Bar = 50 μm.

As PARPi is a chemo-sensitising agent, we examined whether the combination of PARPi and chemotherapy could further reduce LL2 tumour growth *in vivo*. PARPi treatment alone had minimal effect on tumour growth and survival, whereas chemotherapy alone delayed tumour growth and increased MST to 14 days which, when combined with 1 or 2 mg/kg PARPi, further delayed tumour growth and increased MST to 17 and 18 days, respectively (Figure 5A,B). No treatment toxicity was evident, with the PARPi-chemotherapy combination resulting in only slight and reversible body weight loss (Additional file 3: Figure S3).

To determine whether DNA damage, cell death and intratumoural DAB4 binding were altered within LL2 tumours after combination treatment, mice were administered with PARPi and chemotherapy followed 24 h later with biotin-DAB4. Mice were euthanized 24 h later and tumours were analysed for DNA damage, cell death and DAB4 binding. DNA damage evident as DSB marked by γ-H2AX foci increased after chemotherapy, and increased further when PARPi was combined with chemotherapy (Figure 5C). Chemotherapy also significantly increased tumour cell death, with the combination of PARPi and chemotherapy resulting in the greatest amount of cell death (Figure 5D). Tumour DAB4 binding was commensurate with treatment-induced cell death (Figure 5E), due in part to DAB4 binding to dead tumour cells (Figure 5F).

### Triple combination of PARPi, chemotherapy and ^177^Lu-DAB4

**Figure 6:**
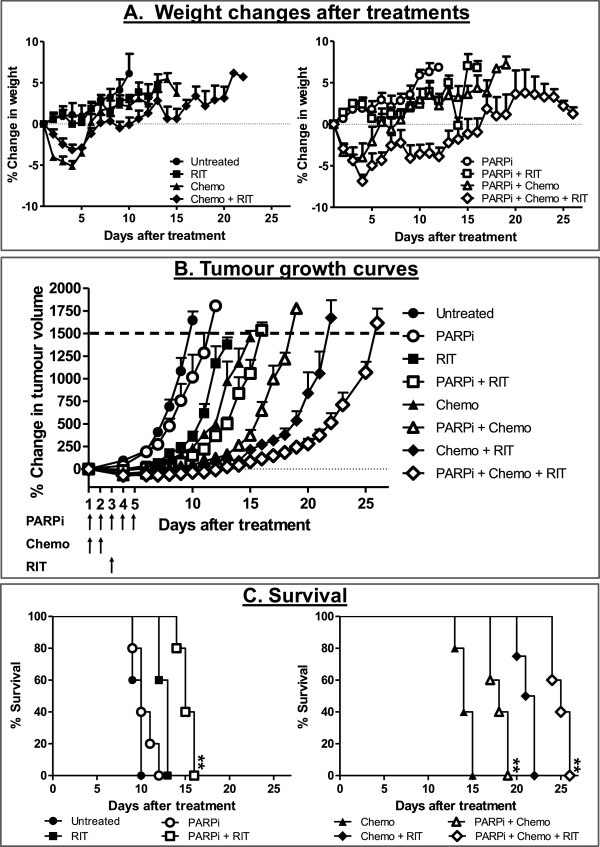
**PARP inhibition potentiates the anti-tumour effects of individual and combination treatments with chemotherapy and RIT.** LL2 tumour-bearing mice were treated with PARPi on days 1 to 5, chemotherapy on days 1 and 2 and 5 MBq ^177^Lu-DAB4 on day 3. **(A)** Weight changes of mice after treatment. **(B)** Percentage change in tumour volume after treatment. **(C)** Kaplan-Meier survival analysis. ** *p* < 0.01 compared to same treatment group but without PARPi administration.

We next examined whether administering ^177^Lu-DAB4 in combination with PARPi and chemotherapy could further potentiate the anti-tumour response. The combination of PARPi with chemotherapy, ^177^Lu-DAB4 or the triple combination of PARPi, chemotherapy and ^177^Lu-DAB4 were well tolerated with only transient and reversible weight loss observed after the triple combination (Figure 6A), with no evident physical signs of distress or discomfort. Combining PARPi with 5 MBq ^177^Lu-DAB4 or chemotherapy increased tumour growth delay (Figure 6B) and significantly improved survival of mice (MST of 15 and 18 days for mice treated with PARPi and RIT or chemotherapy, respectively) compared to the equivalent treatment without the addition of PARPi (MST of 13 and 14 days for mice treated with RIT or chemotherapy alone; Figure 6C). The triple combination of chemotherapy, PARPi and ^177^Lu-DAB4 produced the greatest anti-tumour response, with a significant increase in survival (MST of 25 days) compared to mice which received only chemotherapy and 5 MBq ^177^Lu-DAB4 (MST of 21 days). Analysis of the TDT for each treatment group demonstrated that the addition of PARPi to the treatment regimens of RIT, chemotherapy or combination of chemotherapy and RIT resulted in an increase in TDT greater than would be expected if the addition of PARPi was only additive (Additional file 4: Figure S4).

## Discussion

The La antigen represents a suitable target for RIT as it is highly abundant and over-expressed at both the mRNA and protein level in malignant human cell cultures [10,31] and in primary human cancers [8,9,31]. Moreover, over-expression of La mRNA portends a worse prognosis in surgically resected NSCLC [27]. La is normally located within the nucleus where it protects nascent RNA from exonucleases reviewed by [32], making it inaccessible to antibody binding. During cell death, La is redistributed to the cytoplasm through protease-mediated cleavage of its C-terminal nuclear localisation signal [33]. This, and the loss of cell membrane integrity during cell death, make La accessible to DAB4 binding and means that DAB4 preferentially binds to dead tumour cells [10]. This was evident as DAB4 only bound to treatment-induced dead LL2 cells and did not bind to viable LL2 cells.

As a monotherapy, ^177^Lu-DAB4 showed significant anti-tumour activity, with the response to 7.5 and 10 MBq doses of ^177^Lu-DAB4 being comparable to chemotherapy. The similar tumour responses to 7.5 and 10 MBq doses (corresponding to approximately 75 and 100 μg of mAb per mouse, respectively) suggest that a saturating dose for ^177^Lu-DAB4 monotherapy had been reached, perhaps because of a limiting number of DAB4-binding dead tumour cell targets [25]. The combination of chemotherapy with ^177^Lu-DAB4 resulted in a supra-additive anti-tumour response, and reflected the similar supra-additive response observed with combined chemotherapy and ^90^Y-DAB4, which we characterised as a ‘genotoxic chain reaction’ [11]. Moreover, ^90^Y-DAB4 and ^177^Lu-DAB4 behaved as residualizing radioimmunoconjugates after combination chemotherapy and RIT (chemo-RIT): intratumoural, detergent-resistant ^90^Y-DAB4 was found 96 h post-chemo-RIT [11], and ^177^Lu-DAB4 was found in LL2 tumours 24 h post-chemo-RIT. The complex mechanism involved in the supra-additive responses may depend on at least two factors:

(i) increased chemotherapy-induced tumour cell death with the associated increase in intratumoural binding of ^177^Lu-DAB4 results in radiation crossfire as ^177^Lu-generated β-particles can kill cells up to 50 cell diameters from antibody-bound target cells [34], thus generating ever more dead cell targets;

(ii) the newly generated dead tumour cells provide additional targets for circulating ^177^Lu-DAB4 to bind thereby permitting continued irradiation of surrounding viable tumour cells.

Furthermore, the MTD of ^177^Lu-DAB4 alone or with chemotherapy was higher than that observed for ^90^Y-DAB4 (MTD of 5 MBq) in the LL2 tumour model [11]. As both ^177^Lu and ^90^Y are used for clinical RIT, we suggest that ^177^Lu may have combined safety and efficacy advantages over ^90^Y as a therapeutic β-emitter, at least in treating small tumour volumes [35]. β-particles derived from the decay of ^177^Lu *vs*^90^Y have a lower maximum energy (0.5 MeV with 79% occurrence *vs* 2.2 MeV with 99% occurrence), lower dose-rate constant (0.076 and 0.54 Gy.g/MBq.h), shorter range (maximum tissue penetration 2 mm *vs* 11 mm), but a longer half-life (6.71 days *vs* 2.67 days). Therefore, the likelihood that the radiation dose absorbed by susceptible tissues such as gut and bone marrow will be lower and that more radiation dose will be absorbed for longer within the confines of the shrinking tumour favours ^177^Lu-DAB4 rather than ^90^Y-DAB4 for La-targeted RIT post-chemotherapy. Consequently, we introduce the concept of ‘volume-adapted radioimmunotherapy’ to describe the match between the physical characteristics of ^177^Lu and tumour size post-chemotherapy treatment.

As the combination of PARPi with chemotherapy or radiotherapy has been shown to have greater potency than each individual treatment [18], we reasoned that the combination of PARPi with chemotherapy should exhibit greater anti-tumour activity than chemotherapy alone. Indeed, combining PARPi with chemotherapy showed greater anti-tumour activity than chemotherapy alone which was due, in part, to an increase in DNA damage and cell death within the tumour. PARPi also potentiated the anti-tumour response to RIT. Although outside the scope of this study, PARPi have been reported to increase autophagy and reduce proliferation within tumours especially when combined with radiation [18], and these mechanisms could also be involved in the anti-tumour responses observed here. However, the greatest anti-tumour response was observed with the triple combination of PARPi, chemotherapy and RIT. This unique combination approach depends entirely on β-radiation crossfire for its therapeutic effectiveness as DAB4 alone has no anti-tumour activity [11]. Therefore, we argue that the addition of a PARP inhibitor to chemo-RIT creates more necrotic tumour cell targets for DAB4 binding and further increases tumour shrinkage, which together enhance the potency of La-directed and volume-adapted RIT.

Nevertheless, tumours were not eradicated after a single cycle of triple combination therapy. Among the possible explanations is that the LL2 cell line is known to have mutant *p53*[36], and altered *p53* expression reduces sensitivity to PARPi [37]. Hence, other radio-sensitising agents such as anti-EGFR mAb may be used to augment chemo-RIT. For example, a neutralising mouse anti-EGFR mAb, which alone displays anti-tumour activity in the Lewis Lung carcinoma model [38], could be added to the treatment regimen to radio-sensitise tumour cells as well as directly inhibit tumour growth. Supporting evidence for this approach is found in the only other known study of the triple combination of PARPi, chemotherapy and RIT (using ^177^Lu-labeled anti-human EGFR mAb). Here, complete eradication of pulmonary metastases and patient-derived xenografts of human triple-negative breast cancer was achieved in immunodeficient mice. This effect of triple therapy was attributed to an increase in tumour cell death and killing of putative breast cancer stem cells [39], which may have been aided by EGFR-mediated inhibition of DNA DSB repair, particularly in PARPi-sensitised breast cancer cells.

## Conclusion

In conclusion, using RIT with ^177^Lu as the payload, we have shown that La-targeted RIT is well tolerated and able to inhibit growth of LL2 tumour syngrafts pre-treated with chemotherapy. Moreover, the addition of a PARPi potentiated the anti-tumour effects of both chemotherapy and RIT. Additional approaches such as repeated cycles of RIT, conjugation to α-emitting radionuclides, or chemo-RIT combinations with inhibitors of EGFR signalling, cell cycle checkpoints, or DNA repair, may contribute to eradication of LL2 tumours. Nonetheless, here we demonstrate the principle that bystander killing by a short-range β-emitter is a feasible and active approach to the treatment of lung cancer. This approach may find clinical application as consolidation treatment for such standard treatments as platinum-based chemotherapy and radiotherapy in advanced disease and adjuvant settings.

## Competing interests

MP Brown, F Al-Ejeh and JM Darby are co-inventors on APOMAB-related patents both issued and pending.

## Authors’ contributions

AHS designed and performed experiments, collected the data, performed statistical analysis and wrote the paper. FA designed and performed experiments, collected the data and performed statistical analysis. CKF and JMD were involved with *in vivo* experiments and data collection. DMR performed data analysis comparing survival of NSCLC patients with La expression and performed statistical analysis. AR analysed the Tissue microarray and JM developed and performed immunohistochemistry and imaging of the Tissue microarray. MPB participated in the design of the study, data collection, analysis and interpretation, and in writing the manuscript. All authors read and approved the final manuscript.

## Supplementary Material

Additional file 1: Figure S1 Mouse body weight change after treatment with RIT alone or in combination with chemotherapy. Figure S1 Mice were treated with RIT alone or with chemotherapy (chemo) as described in Methods. The percent change in mouse weights for mice treated with RIT alone **(A)** or chemotherapy and RIT **(B)** are shown, *n* = 5. Click here for file

Additional file 2: Figure S2 LL2 tumour doubling time after treatment with ^177^Lu-DAB4 or ^90^Y-DAB4 alone or in combination with chemotherapy. Figure S2 Mean tumour doubling times (TDT) for tumour-bearing mice treated with **(A) **^177^Lu-DAB4 or **(B) **^90^Y-DAB4 alone or combined with chemotherapy were derived from tumour growth curves. The broken line represents the TDT if the combination of chemotherapy and RIT were additive. Data from **(B)** has been published previously in [11]. Click here for file

Additional file 3: Figure S3 Mouse body weight changes after treatment. Figure S3 Mice were treated with PARPi inhibitor (1 or 2 mg/kg) alone or with chemotherapy (chemo) as described in Methods. The percent change in mouse weights for mice treated with PARPi alone **(A)** or PARPi and chemotherapy **(B)** are shown, *n* = 4–5. Click here for file

Additional file 4: Figure S4 LL2 tumour doubling time after treatment regimens including PARPi, chemotherapy or RIT. Figure S4 Mean tumour doubling times (TDT) for tumour-bearing mice in each treatment group were derived from tumour growth curves. Filled bars indicate increases in TDT that would be expected from adding the effects of component treatments, whereas open bars indicate the observed TDT for treatments. Additive effects were calculated by deducting the TDT of the untreated group from the TDT of the PARPi treatment group and adding that to the TDT of the group of interest, e.g. TDT_PARPi + RIT_ = TDT_RIT_ + (TDT_PARPi_ – TDT_untreated_). Click here for file

## References

[B1] Jemal A Bray F Center MM Ferlay J Ward E Forman D Global cancer statistics CA Cancer J Clin 2011 4 69 90 10.3322/caac.20107 21296855

[B2] Li T Kung HJ Mack PC Gandara DR Genotyping and genomic profiling of non-small-cell lung cancer: implications for current and future therapies J Clin Oncol 2013 4 1039 1049 10.1200/JCO.2012.45.3753 23401433 PMC3589700

[B3] Oxnard GR Binder A Janne PA New targetable oncogenes in non-small-cell lung cancer J Clin Oncol 2013 4 1097 1104 10.1200/JCO.2012.42.9829 23401445 PMC3589703

[B4] Pignon JP Tribodet H Scagliotti GV Douillard JY Shepherd FA Stephens RJ Dunant A Torri V Rosell R Seymour L Spiro SG Rolland E Fossati R Aubert D Ding K Waller D Le Chevalier T LACE Collaborative Group Lung adjuvant cisplatin evaluation: a pooled analysis by the LACE Collaborative Group J Clin Oncol 2008 4 3552 3559 10.1200/JCO.2007.13.9030 18506026

[B5] Enger SA Hartman T Carlsson J Lundqvist H Cross-fire doses from beta-emitting radionuclides in targeted radiotherapy. A theoretical study based on experimentally measured tumor characteristics Phys Med Biol 2008 4 1909 1920 10.1088/0031-9155/53/7/007 18364546

[B6] Kovtun YV Audette CA Ye Y Xie H Ruberti MF Phinney SJ Leece BA Chittenden T Blättler WA Goldmacher VS Antibody-drug conjugates designed to eradicate tumors with homogeneous and heterogeneous expression of the target antigen Cancer Res 2006 4 3214 3221 10.1158/0008-5472.CAN-05-3973 16540673

[B7] Song H Sgouros G Radioimmunotherapy of solid tumors: searching for the right target Curr Drug Deliv 2011 4 26 44 10.2174/156720111793663651 21034423 PMC4337879

[B8] Trotta R Vignudelli T Candini O Intine RV Pecorari L Guerzoni C Santilli G Byrom MW Goldoni S Ford LP Caligiuri MA Maraia RJ Perrotti D Calabretta B BCR/ABL activates mdm2 mRNA translation via the La antigen Cancer Cell 2003 4 145 160 10.1016/S1535-6108(03)00020-5 12620409

[B9] Sommer G Rossa C Chi AC Neville BW Heise T Implication of RNA-binding protein La in proliferation, migration and invasion of lymph node-metastasized hypopharyngeal SCC cells PLoS ONE 2011 4 e25402 10.1371/journal.pone.0025402 22016766 PMC3189910

[B10] Al-Ejeh F Darby JM Brown MP The La autoantigen is a malignancy-associated cell death target that is induced by DNA-damaging drugs Clin Cancer Res 2007 4 5509s 5518s 10.1158/1078-0432.CCR-07-0922 17875783

[B11] Al-Ejeh F Darby JM Brown MP Chemotherapy synergizes with radioimmunotherapy targeting la autoantigen in tumors PLoS ONE 2009 4 e4630 10.1371/journal.pone.0004630 19247485 PMC2645682

[B12] Al-Ejeh F Darby JM Tsopelas C Smyth D Manavis J Brown MP APOMAB, a La-specific monoclonal antibody, detects the apoptotic tumor response to life-prolonging and DNA-damaging chemotherapy PLoS ONE 2009 4 e4558 10.1371/journal.pone.0004558 19247492 PMC2645692

[B13] Al-Ejeh F Brown MP Speer TW Combined Modality Therapy: Relevance for Targeted Radionuclide Therapy Targeted Radionuclide Therapy 2011 1 Philadelphia: Lippincott, Williams & Wilkinson 220 235

[B14] Bartkova J Horejsi Z Koed K Kramer A Tort F Zieger K Guldberg P Sehested M Nesland J Lukas C Ørntoft T Lukas J Bartek J DNA damage response as a candidate anti-cancer barrier in early human tumorigenesis Nature 2005 4 864 870 10.1038/nature03482 15829956

[B15] Al-Ejeh F Kumar R Wiegmans A Lakhani SR Brown MP Khanna KK Harnessing the complexity of DNA-damage response pathways to improve cancer treatment outcomes Oncogene 2010 4 6085 6098 10.1038/onc.2010.407 20818418

[B16] Sousa FG Matuo R Soares DG Escargueil AE Henriques JA Larsen AK Safi J PARPs and the DNA damage response Carcinogenesis 2012 4 1433 1440 10.1093/carcin/bgs132 22431722

[B17] Javle M Curtin NJ The role of PARP in DNA repair and its therapeutic exploitation Br J Cancer 2011 4 1114 1122 10.1038/bjc.2011.382 21989215 PMC3208503

[B18] Albert JM Cao C Kim KW Willey CD Geng L Xiao D Wang H Sandler A Johnson DH Colevas AD Low J Rothenberg ML Lu B Inhibition of poly(ADP-ribose) polymerase enhances cell death and improves tumor growth delay in irradiated lung cancer models Clin Cancer Res 2007 4 3033 3042 10.1158/1078-0432.CCR-06-2872 17505006

[B19] Ali M Telfer BA McCrudden C O'Rourke M Thomas HD Kamjoo M Kyle S Robson T Shaw C Hirst DG Curtin NJ Williams KJ Vasoactivity of AG014699, a clinically active small molecule inhibitor of poly(ADP-ribose) polymerase: a contributory factor to chemopotentiation in vivo? Clin Cancer Res 2009 4 6106 6112 10.1158/1078-0432.CCR-09-0398 19789326 PMC2756456

[B20] Senra JM Telfer BA Cherry KE McCrudden CM Hirst DG O'Connor MJ Wedge SR Stratford IJ Inhibition of PARP-1 by olaparib (AZD2281) increases the radiosensitivity of a lung tumor xenograft Mol Cancer Ther 2011 4 1949 1958 10.1158/1535-7163.MCT-11-0278 21825006 PMC3192032

[B21] Calabrese CR Almassy R Barton S Batey MA Calvert AH Canan-Koch S Durkacz BW Hostomsky Z Kumpf RA Kyle S Li J Maegley K Newell DR Notarianni E Stratford IJ Skalitzky D Thomas HD Wang LZ Webber SE Williams KJ Curtin NJ Anticancer chemosensitization and radiosensitization by the novel poly(ADP-ribose) polymerase-1 inhibitor AG14361 J Natl Cancer Inst 2004 4 56 67 10.1093/jnci/djh005 14709739

[B22] Calabrese CR Batey MA Thomas HD Durkacz BW Wang LZ Kyle S Skalitzky D Li J Zhang C Boritzki T Maegley K Calvert AH Hostomsky Z Newell DR Curtin NJ Identification of potent nontoxic poly(ADP-Ribose) polymerase-1 inhibitors: chemopotentiation and pharmacological studies Clin Cancer Res 2003 4 2711 2718 12855651

[B23] Ali M Kamjoo M Thomas HD Kyle S Pavlovska I Babur M Telfer BA Curtin NJ Williams KJ The clinically active PARP inhibitor AG014699 ameliorates cardiotoxicity but does not enhance the efficacy of doxorubicin, despite improving tumor perfusion and radiation response in mice Mol Cancer Ther 2011 4 2320 2329 10.1158/1535-7163.MCT-11-0356 21926192 PMC3242069

[B24] Kremerskothen J Nettermann M op de Bekke A Bachmann M Brosius J Identification of human autoantigen La/SS-B as BC1/BC200 RNA-binding protein DNA Cell Biol 1998 4 751 759 10.1089/dna.1998.17.751 9778034

[B25] Al-Ejeh F Darby JM Pensa K Diener KR Hayball JD Brown MP In vivo targeting of dead tumor cells in a murine tumor model using a monoclonal antibody specific for the La autoantigen Clin Cancer Res 2007 4 5519s 5527s 10.1158/1078-0432.CCR-07-0964 17875784

[B26] Al-Ejeh F Darby JM Thierry B Brown MP A simplified suite of methods to evaluate chelator conjugation of antibodies: effects on hydrodynamic radius and biodistribution Nucl Med Biol 2009 4 395 402 10.1016/j.nucmedbio.2009.01.001 19423007

[B27] Shedden K Taylor JM Enkemann SA Tsao MS Yeatman TJ Gerald WL Eschrich S Jurisica I Giordano T Misek D Chang A Zhu C Strumpf D Hanash S Shepherd F Ding K Seymour L Naoki K Pennell N Weir B Verhaak R Ladd-Acosta C Golub T Gruidl M Sharma A Szoke J Zakowski M Rusch V Kris M Viale A Gene expression-based survival prediction in lung adenocarcinoma: a multi-site, blinded validation study Nat Med 2008 4 822 827 10.1038/nm.1790 18641660 PMC2667337

[B28] Abramson JH WINPEPI (PEPI-for-Windows): computer programs for epidemiologists Epidemiol Perspect Innovat 2004 4 6 10.1186/1742-5573-1-6 PMC544871 15606913

[B29] Landi MT Dracheva T Rotunno M Figueroa JD Liu H Dasgupta A Mann E Fukuoka J Hames M Bergen A Murphy S Yang P Pesatori A Consonni D Bertazzi PA Wacholder S Shih J Caporaso N Je J Gene expression signature of cigarette smoking and its role in lung adenocarcinoma development and survival PLoS ONE 2008 4 e1651 10.1371/journal.pone.0001651 18297132 PMC2249927

[B30] Hou J Aerts J den Hamer B van Ijcken W den Bakker M Riegman P van der Leest C van der Spek P Foekens J Hoogsteden H Philipsen S Gene expression-based classification of non-small cell lung carcinomas and survival prediction PLoS ONE 2010 4 e10312 10.1371/journal.pone.0010312 20421987 PMC2858668

[B31] Sommer G Dittmann J Kuehnert J Reumann K Schwartz PE Will H Coulter BL Smith MT Heise T The RNA-binding protein La contributes to cell proliferation and CCND1 expression Oncogene 2011 4 434 444 10.1038/onc.2010.425 20856207

[B32] Wolin SL Cedervall T The La protein Annu Rev Biochem 2002 4 375 403 10.1146/annurev.biochem.71.090501.150003 12045101

[B33] Ayukawa K Taniguchi S Masumoto J Hashimoto S Sarvotham H Hara A Aoyama T Sagara J La autoantigen is cleaved in the COOH terminus and loses the nuclear localization signal during apoptosis J Biol Chem 2000 4 34465 34470 10913436 10.1074/jbc.M003673200

[B34] Schlom J Siler K Milenic DE Eggensperger D Colcher D Miller LS Houchens D Cheng R Kaplan D Goeckeler W Monoclonal antibody-based therapy of a human tumor xenograft with a 177lutetium-labeled immunoconjugate Cancer Res 1991 4 2889 2896 1851665

[B35] Scott AM Radioimmunotherapy of prostate cancer: does tumor size matter? J Clin Oncol 2005 4 4567 4569 10.1200/JCO.2005.01.903 15837975

[B36] Rizzo MG Soddu S Tibursi G Calabretta B Sacchi A Wild-type p53 differentially affects tumorigenic and metastatic potential of murine metastatic cell variants Clin Exp Metastasis 1993 4 368 376 10.1007/BF00132980 8375112

[B37] Chuang HC Kapuriya N Kulp SK Chen CS Shapiro CL Differential anti-proliferative activities of poly(ADP-ribose) polymerase (PARP) inhibitors in triple-negative breast cancer cells Breast Cancer Res Treat 2012 4 649 659 10.1007/s10549-012-2106-5 22678161 PMC4297209

[B38] Garrido G Rabasa A Sanchez B Lopez MV Blanco R Lopez A Hernández DR Pérez R Fernández LE Induction of immunogenic apoptosis by blockade of epidermal growth factor receptor activation with a specific antibody J Immunol 2011 4 4954 4966 10.4049/jimmunol.1003477 21984704

[B39] Al-Ejeh F Shi W Miranda M Simpson PT Vargas AC Song S Wiegmans AP Swarbrick A Welm AL Brown MP Chenevix-Trench G Lakhani SR Khanna KK Treatment of Triple-Negative Breast Cancer Using Anti-EGFR Directed Radioimmunotherapy Combined with Radiosensitizing Chemotherapy and PARP Inhibitor J Nucl Med 2013 4 913 921 10.2967/jnumed.112.111534 23564760

