# Supplementary material for: The La antigen is over-expressed in lung cancer and is a selective dead cancer cell target for radioimmunotherapy using the La-specific antibody APOMAB®
Source: EJNMMI Res. 2014 Jan 4;4:2. doi: 10.1186/2191-219X-4-2 (PMC3882100; doi:10.1186/2191-219X-4-2)
Supplement: Additional file 1: Figure S1 — Mouse body weight change after treatment with RIT alone or in combination with chemotherapy. Figure S1 Mice were treated with RIT alone or with chemotherapy (chemo) as described in Methods. The percent change in mouse weights for mice treated with RIT alone (A) or chemotherapy and RIT (B) are shown, n = 5. [file 2191-219X-4-2-S1.doc]

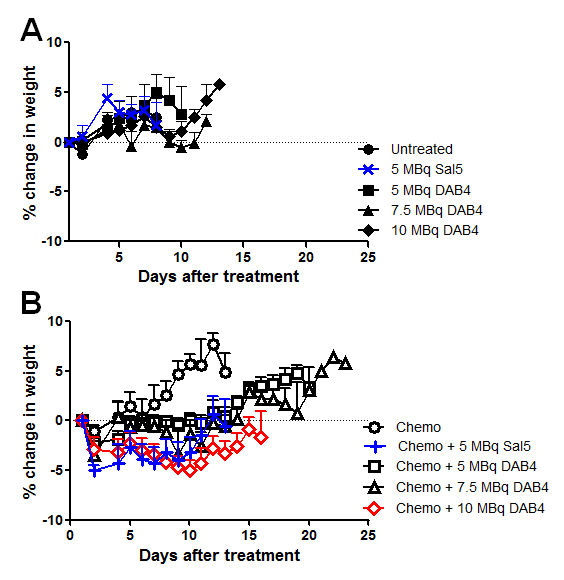


Additional file 1: Figure S1 Mouse body weight changes after treatment with RIT alone or in combination with chemotherapy.

Mice were treated with RIT alone or with chemotherapy (chemo) as described in Materials and Methods. The percent change in mouse weights for mice treated with RIT alone (A) or chemotherapy and RIT (B) are shown, *n* = 5
