# Supplementary material for: The La antigen is over-expressed in lung cancer and is a selective dead cancer cell target for radioimmunotherapy using the La-specific antibody APOMAB®
Source: EJNMMI Res. 2014 Jan 4;4:2. doi: 10.1186/2191-219X-4-2 (PMC3882100; doi:10.1186/2191-219X-4-2)
Supplement: Additional file 2: Figure S2 — LL2 tumour doubling time after treatment with 177Lu-DAB4 or 90Y-DAB4 alone or in combination with chemotherapy. Figure S2 Mean tumour doubling times (TDT) for tumour-bearing mice treated with (A) 177Lu-DAB4 or (B) 90Y-DAB4 alone or combined with chemotherapy were derived from tumour growth curves. The broken line represents the TDT if the combination of chemotherapy and RIT were additive. Data from (B) has been published previously in [11]. [file 2191-219X-4-2-S2.doc]

**A**

**B**

Additional file 2: Figure S2 LL2 tumour doubling time after treatment with ^177^Lu-DAB4 or ^90^Y-DAB4 alone or in combination with chemotherapy.

Mean tumour doubling times (TDT) for tumour-bearing mice treated with (A) ^177^Lu-DAB4 or (B) ^90^Y-DAB4 alone or combined with chemotherapy were derived from tumour growth curves. The broken line represents the TDT if the combination of chemotherapy and RIT were additive. Data from (B) has been published previously in [11].
