# Supplementary material for: The La antigen is over-expressed in lung cancer and is a selective dead cancer cell target for radioimmunotherapy using the La-specific antibody APOMAB®
Source: EJNMMI Res. 2014 Jan 4;4:2. doi: 10.1186/2191-219X-4-2 (PMC3882100; doi:10.1186/2191-219X-4-2)
Supplement: Additional file 3: Figure S3 — Mouse body weight changes after treatment. Figure S3 Mice were treated with PARPi inhibitor (1 or 2 mg/kg) alone or with chemotherapy (chemo) as described in Methods. The percent change in mouse weights for mice treated with PARPi alone (A) or PARPi and chemotherapy (B) are shown, n = 4–5. [file 2191-219X-4-2-S3.doc]

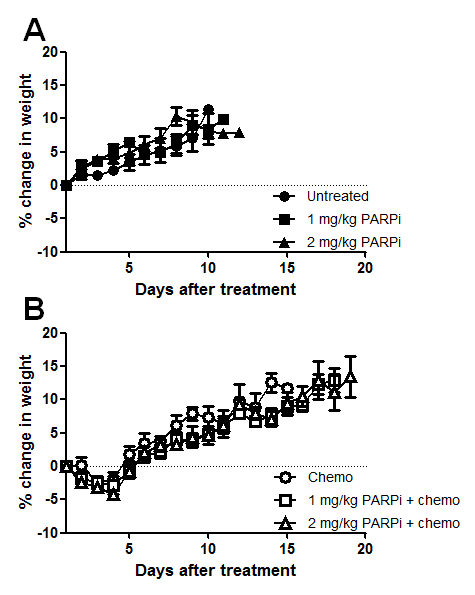


Additional file 3: Figure S3 Mouse body weight changes after treatment.

Mice were treated with PARPi inhibitor (1 or 2 mg/kg) alone or with chemotherapy (chemo) as described in Materials and Methods. The percent change in mouse weights for mice treated with PARPi alone (A) or PARPi and chemotherapy (B) are shown, *n* = 4-5
