# Supplementary material for: The La antigen is over-expressed in lung cancer and is a selective dead cancer cell target for radioimmunotherapy using the La-specific antibody APOMAB®
Source: EJNMMI Res. 2014 Jan 4;4:2. doi: 10.1186/2191-219X-4-2 (PMC3882100; doi:10.1186/2191-219X-4-2)
Supplement: Additional file 4: Figure S4 — LL2 tumour doubling time after treatment regimens including PARPi, chemotherapy or RIT. Figure S4 Mean tumour doubling times (TDT) for tumour-bearing mice in each treatment group were derived from tumour growth curves. Filled bars indicate increases in TDT that would be expected from adding the effects of component treatments, whereas open bars indicate the observed TDT for treatments. Additive effects were calculated by deducting the TDT of the untreated group from the TDT of the PARPi treatment group and adding that to the TDT of the group of interest, e.g. TDTPARPi + RIT = TDTRIT + (TDTPARPi – TDTuntreated). [file 2191-219X-4-2-S4.doc]

Additional file 4: Figure S4 LL2 tumour doubling time after treatment regimens including PARPi, chemotherapy or RIT.

Mean tumour doubling times (TDT) for tumour-bearing mice in each treatment group were derived from tumour growth curves. Filled bars indicate increases in TDT that would be expected from adding the effects of component treatments, whereas open bars indicate the observed TDT for treatments. Additive effects were calculated by deducting the TDT of the untreated group from the TDT of the PARPi treatment group and adding that to the TDT of the group of interest, e.g. TDT_PARPi + RIT_ = TDT_RIT_ + (TDT_PARPi_ – TDT_untreated_).
